# Psychobiotic Supplementation of PS128^TM^ Improves Stress, Anxiety, and Insomnia in Highly Stressed Information Technology Specialists: A Pilot Study

**DOI:** 10.3389/fnut.2021.614105

**Published:** 2021-03-26

**Authors:** Shu-I Wu, Chien-Chen Wu, Pei-Joung Tsai, Li-Hao Cheng, Chih-Chieh Hsu, Ian-Kai Shan, Po-Ying Chan, Ting-Wei Lin, Chih-Jung Ko, Wan-Lin Chen, Ying-Chieh Tsai

**Affiliations:** ^1^Department of Medicine, MacKay Medical College, New Taipei City, Taiwan; ^2^Section of Psychiatry and Suicide Prevention Center, MacKay Memorial Hospital, Taipei, Taiwan; ^3^Bened Biomedical Co., Ltd., Taipei, Taiwan; ^4^Department of Psychiatry, Taipei Veterans General Hospital, Taipei, Taiwan; ^5^Department of Medical Research, Mackay Memorial Hospital, Taipei, Taiwan; ^6^Institute of Biochemistry and Molecular Biology, National Yang-Ming University, Taipei, Taiwan

**Keywords:** probiotic, stress, *Lactobacillus plantarum*, PS128^TM^, psychobiotic

## Abstract

**Background:** Information technology (IT) is an industry related to the production of computers, information processing, and telecommunications. Such industries heavily rely on the knowledge and solutions provided by IT specialists. Previous reports found that the subjective stress scores were higher in IT specialists who developed diabetes, hypertension, and depression. Specific probiotics, known as psychobiotics, may alleviate stress and mood symptoms. This study aimed to examine whether an 8-week intervention of a novel psychobiotic, *Lactobacillus plantarum* PS128^TM^ (PS128^TM^), improved self-perceived stress and mood symptoms among high-stress IT specialists.

**Methods:** This open-label, single-arm, baseline-controlled study included IT specialists from a large IT company in Northern Taiwan. Participants with a Perceived Stress Scale (PSS) 10-item version score of 27 or higher were included. Participants were asked to take two capsules containing PS128^TM^ powder, equivalent to 20 billion colony-forming units, daily. Self-report measures, such as the Job Stress Scale, Visual Analog Scale of Stress, the Insomnia Severity Index, the State and Trait Anxiety Index, the Questionnaire for Emotional Trait and State, the Patient Health Questionnaire, the Quality of Life Enjoyment and Satisfaction Questionnaire, and Gastrointestinal Severity Index were compared at baseline and at the end of the trial period. The primary outcome was a 20% reduction in the PSS score at endpoint. Objective measures included salivary levels of stress biomarkers, including cortisol, α-amylase, immunoglobulin A, lactoferrin, and lysozymes, as well as results of the Test of Attentional Performance.

**Results:** Of the 90 eligible IT specialists, 36 met the inclusion criteria. After the 8-week trial period, significant improvements in self-perceived stress, overall job stress, job burden, cortisol level, general or psychological health, anxiety, depression, sleep disturbances, quality of life, and both positive and negative emotions were found.

**Conclusion:** Our results suggest that PS128^TM^ has the distinct advantage of providing stress relief and can improve mental health for people with a high-stress job. Future placebo-controlled studies are warranted to explore the effect and underlying mechanisms of action of PS128^TM^.

**Clinical Trial Registration:** https://clinicaltrials.gov/ (identifier: NCT04452253-sub-project 2).

## Introduction

Information technology (IT) is an industry sector related to computer processing. It involves the development of computer hardware, software, semiconductors, websites, applications of statistical methods for decision-making, or telecommunications that encompass transmitting information or systems that facilitate communication through computer programs (1). In the past two decades, the number of people employed in the IT industry has grown rapidly in the US, from 400,000 people in 1990 to 1,800,000 people in 2011 (2). With an output value of 47 billion US dollars (3), the IT industry in Taiwan is ranked fourth in the world, and accounts for 3.3% of Taiwan's overall employment (4) and 92.8% of exported products. Working in the IT sector usually requires extensive knowledge and the ability to perform complex tasks to develop products or provide services and efficient solutions within limited time frames (5). Previous studies have reported that up to 54% of IT workers may have elevated stress, depression, anxiety, or sleep problems due to the high job demand and the uncertainty in their working environment (6).

When an individual is under chronic stress, their sleep and psychological well-being is often impaired (7). Research has found positive correlations between sleep disturbances and higher levels of perceived stress or job demands in high stress workers such as nurses or shift workers (8). Persistent impairment in sleep function may also augment perceived stress and work-related burnout (9). Long-term poor sleep and high stress at work may be related to the increased prevalence of anxiety and depression (10). Sleep deprivation and circadian rhythm changes might also increase cortisol levels, which in turn affects the circadian rhythm through activation of the hypothalamus-pituitary-adrenal (HPA) axis (11).

Microorganisms and their metabolites regulate the body through a series of biochemical and functional linkages. Growing evidence has demonstrated that the intestinal microbiota and their metabolites play key roles in absorption, metabolism, and maintenance of healthy immune and brain function, and may influence host behaviors (12, 13). Physiological and psychological stress may damage the intestinal microbiota and affect intestinal permeability, leading to poor health (14). Intestinal dysbiosis was shown to be associated with dysregulation of the hippocampal serotonergic systems and the induction of anxiety behaviors in mice models (15). Psychobiotics, a class of probiotics, are defined as live microorganisms that, when ingested in adequate amounts, induces health benefits in patients with psychiatric illness (16). A previous study found small, but significant pooled effects of probiotics on depression (*d* = −0.24, *p* < 0.01) (17) and anxiety (*d* = −0.10, *p* = 0.03) (18). However, no significant differences in the levels of subjective stress or sleep qualities were found in some probiotic formulations among healthy volunteers when compared to the placebo (19, 20).

Previously, *Lactobacillus plantarum* PS128^TM^ (PS128^TM^), isolated from spontaneously fermented mustard greens in Taiwan (21), has been demonstrated to reduce anxiety- and depression-like behaviors (22, 23), ameliorate tic-like behaviors (24), and improve visceral hypersensitivity (25) in experimental animals. Beneficial effects of PS128^TM^ on children with autism spectrum disorder (26), triathletes (27), and patients with major depressive disorder (28) have also been reported. PS128^TM^ may exert its psychotropic effects by altering monoamine neurotransmitter levels (22–25), and modulating glucocorticoid (22), anti-inflammatory, and anti-oxidative responses (21, 22, 27); however, the underlying mechanisms warrant further investigation. Despite the increase in the number of IT employees, the importance of the IT industry to the economy, and the high stress due to uncertainty and workload under time pressure, no study to date has investigated whether probiotics could help alleviate stress, sleep, or mood symptoms in highly stressed IT specialists. Hence, embedded within a large ongoing clinical trial that contained two sub-projects investigating possible effects of PS128 ^TM^ on high stress workers (sub-project 1 on registered nurses, and sub-project 2 on IT specialists), this study aimed to examine whether an 8-week probiotic intervention improved self-perceived stress and job-related stress, symptoms of anxiety or depression, and insomnia severity among highly stressed IT specialists. Furthermore, correlations between all psychological measures and stress biomarkers in IT specialists with high self-perceived stress were also assessed.

## Materials and Methods

### Study Design and Participants

We conducted this open-label study to examine the effects of 8-week supplementation of PS128^TM^ among highly stressed information technology (IT) specialists within the framework of a larger clinical trial (NCT04452253). In the clinical registration of NCT04452253, a randomized, placebo-controlled trial targeted on registered nurses (sub-project 1); and the current open-label single arm study targeted on IT specialists (sub-project 2) were included. Although the overall trial aimed and hypothesized that the 8-weeks intervention of PS128 ^TM^ may reduce perceived stress and associated mood symptoms among high stress workers including registered nurses or IT specialists, these were two separate and independent sub-projects, i.e., the study designs, outcome measures, and target participants were all different. The sub-project 1 randomized placebo-controlled trial aimed to examine the efficacies of psychobiotics PS128^TM^, PS23^TM^, and the PS23^TM^ heat-treated capsule, on reducing stress and mood symptoms among high stress registered nurses, compared placebo. The sub-project 2 open-label single arm study was designed to investigate the tolerability and safety of PS128 among high-stress IT specialists. Herein, we report our findings from the sub-project 2 single arm study that focused on examining the safety and tolerability of PS128^TM^ among IT specialists. Participants were recruited from a large IT company in Northern Taiwan. After written consent was signed, all IT specialists aged 20–60 years (*n* = 90) from this company were screened by the 10-item version of the Perceived Stress Scale (PSS-10). Those with a total PSS-10 score of 27 or higher were invited to participate in this study. Participants were excluded if they had taken or were taking antibiotics or psychotropics within the preceding month, used or were currently using any powder, capsule, or tablet probiotic products (except yogurts) within the preceding 2 weeks, had undergone surgery for any hepatobiliary gastrointestinal disorders, had any past or present inflammatory bowel disease, had a history of cancer, were allergic to lactic acid bacteria, were currently pregnant or breastfeeding, or those who smoked, consumed alcohol, or chewed betel nuts. Participants withdrew from the study if adverse reactions (such as diarrhea or bloating) occurred or if they were no longer willing to participate.

IT specialists with PSS-10 scores of ≥27 were further evaluated by psychological measures and tests on attentional performance at baseline (Time 1) and 8 weeks after the intervention (Time 2). Saliva was collected between 9 a.m. and 12 p.m. at baseline and at the end of 8 weeks to minimize the impact of the circadian rhythm. The subjects were asked not to drink, eat, or brush their teeth at least 1 h before the sample collection. In addition, participants were asked to not perform any kind of exercise within 4 h before saliva collection, and to not consume alcohol 12 h before sampling. Saliva was collected using a commercial device, Cortisol-Salivette® (Sarstedt, Nümbrecht, Germany). After collection, the saliva was extracted by centrifugation for 10 min at 1,000 × g and frozen at −80°C until analysis. After completing the baseline evaluation, two PS128^TM^ capsules were administered. Participants were then asked to take two capsules the next day before bedtime (Time 1), and once daily for 8 weeks. Participants were asked to return the remaining capsules at Time 2 to record the number of capsules consumed. This study was approved by the Institutional Review Board of Mackay Memorial Hospital (IRB no: 19CT013be).

### Materials

The psychobiotic capsule contains 300 mg of lyophilized PS128^TM^ powder, which is equivalent to 10 billion colony forming units of PS128^TM^. It has been approved as a food supplement by the Taiwan Food and Drug Administration, and toxicological assessments suggest that PS128^TM^ is safe for human consumption (29).

### Perceived Stress Scale

The primary outcome was differences in the PSS score before and after consumption PS128^TM^. The PSS is the most widely used psychological tool for measuring how perceived stress affects the participant's feelings within the past month (30). Past evidence regarding its effectiveness in research applications demonstrated that higher PSS scores were associated with failure to quit smoking (31), inability to control blood sugar levels among patients with diabetes mellitus (32), and increased susceptibility to depression or anxiety (33). A PSS-10 score between 27 and 40 indicates high perceived stress (34). The Mandarin version of the PSS was translated and was shown to have good validity, and was used in this study (35).

### The State and Trait Anxiety Index

Differences in the STAI before and after the consumption of PS128^TM^ were compared. The STAI contains two subscales to measure levels of anxiety in the current “state” or from the “trait.” Higher STAI scores are correlated with higher levels of anxiety (36). The Mandarin version of STAI was found to have good reliability, and was used in this study (37).

### Questionnaire of Emotional Trait and State

This scale was used to evaluate the positive and negative effects of PS128^TM^ intervention. According to a principal component analysis, this 36-item questionnaire can be categorized into four different domains of emotions: happiness and acceptance, sadness and being scared, anger and disgust, and hopefulness. This questionnaire was reported to have good internal consistency (Cronbach's α = 0.93) and construct validity (38).

### Patient Health Questionnaire-9

The PHQ-9 was used to screen and monitor the participant's level of depression. The Chinese version of the PHQ-9 can be used to detect depression in participants with a total score of ≥10, with a high sensitivity of 0.86 (39).

### Insomnia Severity Index

The ISI is composed of seven questions to measure sleep difficulties. In patients under primary care, a total score of ≥14 indicated insomnia, with an area under receiver operating curve of 0.87, which demonstrated good validity (40).

### Quality of Life Enjoyment and Satisfaction Questionnaire Short Form

The Q-LES-Q SF contains questions related to physical health, activity, general well-being, relaxation, work function, housework, school work, leisure activities, and social relations in the past week (41). The higher the score, the higher the participant's level of satisfaction. The Mandarin version of Q-LES-Q SF was reported to have good reliability and validity (42).

### Job Stress Scale

The Chinese version of the JSS (43) was translated by the Ministry of Labor in Taiwan. It contains 38 items to evaluate the degree of self-perceived stress caused by the job, satisfaction, interpersonal relationships, and sense of well-being (44). We used the JSS under the hypothesis that the job burden in the specialists' work environment did not significantly change before and after the intervention.

### Visual Analog Scale of Gastrointestinal Symptoms

Differences in 10 gastrointestinal symptoms, including dry mouth, difficulty swallowing, decreased appetite, nausea or vomiting, flatulence, gastralgia, upper and lower abdominal pain, constipation, and diarrhea (45, 46), before and after the intervention were compared to determine the safety and tolerability of the intervention.

### The Visual Analog Scale of Occupational Stress

The VAS was applied for assessing occupational stress. A VAS score of 7.0 has been reported to have a good sensitivity of 0.74 and specificity of 0.93 when compared to the PSS to detect high-stress in workers (47). Differences in VAS scores before and after PS128^TM^ consumption were compared.

### Tests of Attentional Performance

We used the windows-based TAP (including subtests of flexibility, working memory, and Go/No go) (48) as a neuropsychological test to evaluate whether PS128^TM^ consumption would improve cognitive or attentional performance (49).

### Salivary Biomarkers

We measured salivary cortisol, α-amylase (50), immunoglobulin A (IgA) (51), lactoferrin (52), and lysozyme (51) levels since they were found to be related to stress and anxiety. A recent study showed that the injection of probiotics for 12 weeks significantly reduced the level of salivary cortisol in nine fatigue subjects (53). Salivary α-amylase, IgA, lactoferrin, and lysozyme levels were measured by enzyme-linked immunosorbent assay kits (Immuno-Biological Laboratories, Inc., USA; Germany; and Assaypro LLC, USA). Salivary cortisol levels were determined using an electrochemiluminescence immunoassay kit (Elecsys Cortisol, Roche Diagnostic, Germany). All procedures were performed according to the manufacturer's instructions.

### Statistical Analysis

All data were entered and error-checked by the research team and supervised by researchers experienced in clinical trials and longitudinal studies. Improvements in psychological symptoms and treatment responses were defined as a >20% change in the PSS score. A paired *t*-test was used to examine changes in continuous variables at Time 1 and 2. The missing data from participants who dropped out of the study were treated as the “last observation carried forward.” To explore possible causal relationships, we performed correlation and mediation analyses between cortisol levels and all psychological measures. A significant result was considered when two-tailed *p*-values were <0.05. SPSS version 12.0 was used to perform all analyses.

## Results

### Demographic Information

**Figure 1:**
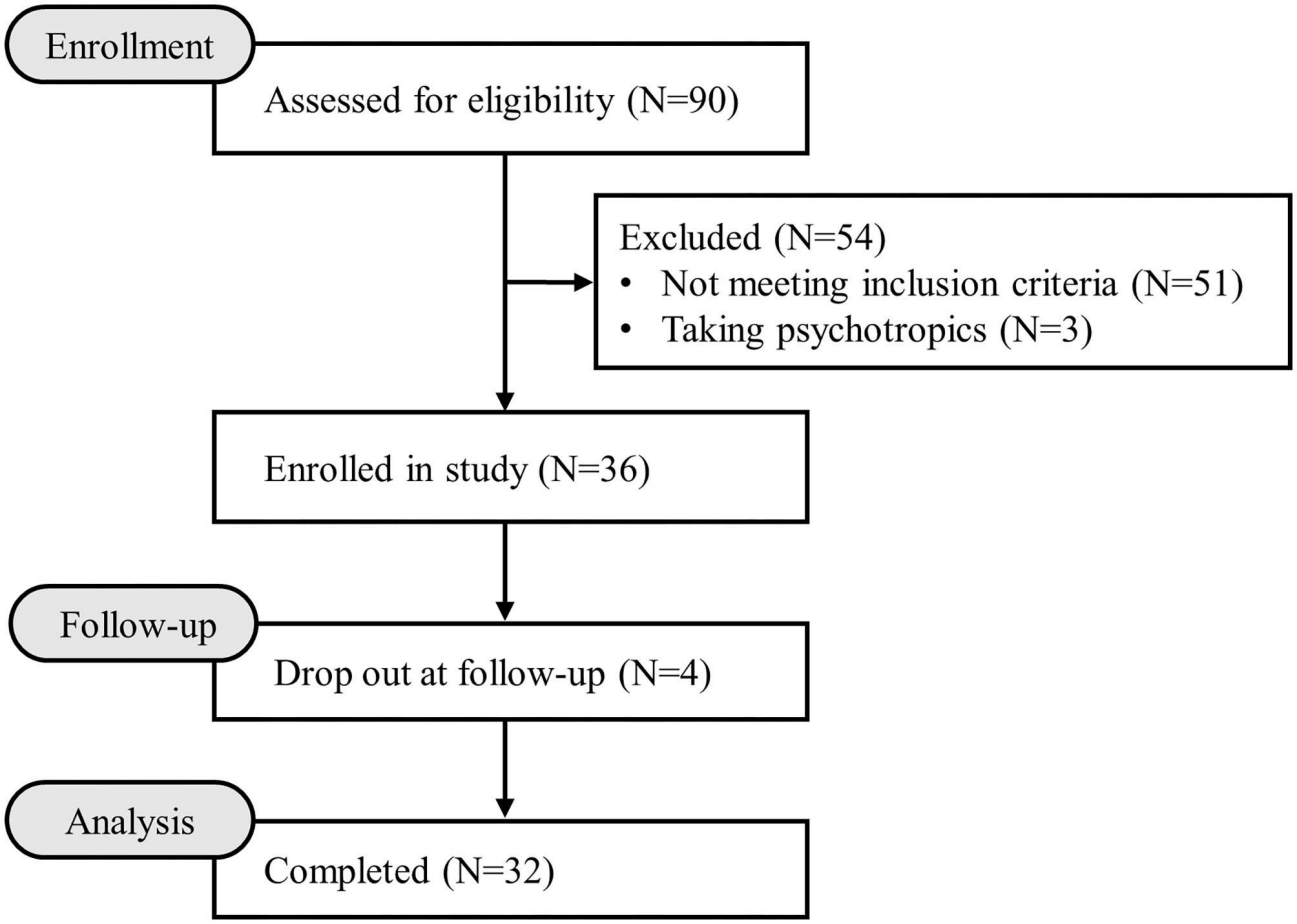
Study flowchart.

As shown in Figure 1, among the 90 IT specialists who provided informed consent, 36 met the inclusion criteria. Of the 36 participants who were included and administered the PS128^TM^ intervention, 32 completed the trial (three dropped out due to inability to return to Taiwan from China due to the pandemic; one dropped out due to self-perceived aggravated sleep condition). Comparisons of the baseline characteristics between participants who enrolled at baseline and those who completed the trial are shown in Table 1. Participants who completed the study had significantly longer work experience than those who enrolled at baseline (*p* = 0.039).

**Table 1:** Comparison of baseline characteristics between participants who enrolled at baseline and those who completed the trial.

	**Baseline participants (*****n*** **=** **36)**		**Trial-completed participants (*****n*** **=** **32)**		***p*-value**
	***n***	**%**	***n***	**%**	
**Gender**					0.303
Female	20	55.56	19	59.38	
Male	16	44.44	13	40.63	
Shift worker	35	97.20	35	96.88	1.000
**Education**					0.593
Senior high + vocational school	5	13.90	4	12.50	
College degree	23	63.90	21	65.63	
Master's degree	8	22.20	7	21.88	
	**Mean**	**SD**	**Mean**	**SD**	***p*****-value**
Age	40.14	8.05	40.34	7.59	0.096
Job experiences (years)	5.56	1.73	5.69	1.64	0.039
Years of education	16.21	1.41	16.16	1.44	0.561
Baseline systolic blood pressure	117.03	14.63	117.31	14.86	0.294
Baseline diastolic blood pressure	79.97	11.70	80.16	11.60	0.392
Baseline body mass index	26.09	7.24	25.59	4.69	0.116

### Subjective Outcomes

The results of the participants' perceived stress and other psychological measures at baseline and after the 8-week trial are shown in Tables 2, 3. Participants showed significant decreases in the levels of self-perceived stress (mean differences: 10.33, SD: 8.12, *p* < 0.001), state and trait anxiety (*p* < 0.001), overall job stress (*p* = 0.003), job burden (*p* = 0.037), insomnia severity (*p* < 0.001), depression (*p* = 0.002), negative emotions (sadness, feeling scared, anger, or disgust), some gastrointestinal symptoms (all *p* < 0.01), and improved mental or physical health (*p* < 0.001). Furthermore, participants showed significant improvements in satisfaction with their quality of life and positive emotions (happiness, acceptance, hopefulness) (all *p* < 0.001). There was no significant difference in blood pressure, but an increase in body weight (mean difference: 0.64 kg, *p* = 0.029) was noted.

**Table 2:** Comparison of the PSS, JSC, ISI, STAI, PHQ, QLESQ, and Emotional Trait and State of participants between the baseline and endpoint.

	**Baseline (*****n*** **=** **36)**		**8 weeks (*****n*** **=** **36; LOCF)**		**Mean differences (Endpoint-baseline)**		*p*-value^*^
	**Mean**	**SD**	**Mean**	**SD**	**Mean differences**	**SD**	
PSS total	34.31	5.39	23.97	6.18	−10.33	8.12	<0.001
**STAI**							
Total	104.92	13.73	93.19	11.90	−11.72	13.29	<0.001
State	50.83	8.47	44.17	7.18	−6.67	8.08	<0.001
Trait	54.08	6.55	49.03	5.70	−5.06	6.06	<0.001
**JSS**							
Job stress	71.67	14.64	63.33	13.09	−8.33	15.40	0.003
Control over job	65.45	9.45	66.44	7.71	0.98	9.11	0.521
Job burden	67.48	11.06	64.29	11.17	−3.18	8.79	0.037
Interpersonal relationships	69.18	12.12	68.66	13.76	−0.52	11.75	0.792
Job satisfaction	60.56	13.93	64.44	12.75	3.89	12.48	0.070
Psychological health	45.89	15.31	55.33	13.27	9.44	14.82	0.001
Energy level	38.19	15.08	51.25	15.60	13.06	14.16	<0.001
General health	60.00	11.90	67.11	10.95	7.11	8.92	<0.001
ISI	12.83	6.07	8.94	4.50	−3.89	4.85	<0.001
PHQ	11.11	5.14	7.78	4.68	−3.33	5.84	0.002
QLESQ	46.42	7.28	50.81	6.84	4.39	6.82	<0.001
**The Questionnaire of Emotional Trait and State**							
Total	89.44	14.09	99.58	11.48	10.14	11.42	<0.001
Happy and acceptance	27.47	4.66	31.28	3.81	3.81	4.14	<0.001
Sad and scared	24.67	4.67	21.33	4.95	−3.33	4.25	<0.001
Angry and disgust	22.36	5.08	20.08	3.81	−2.28	3.93	0.001
Look forward to	11.11	2.03	12.33	1.77	1.22	2.09	0.001

* *Changes between the baseline and endpoint were assessed by a paired t-test*.

*PSS, Perceived Stress Scale; STAI, State Trait Anxiety Index; JSS, Job Stress Scale; ISI, Insomnia Severity Index; PHQ, Patient Health Questionnaire; QLESQ, Quality of Life Enjoyment and Satisfaction Questionnaire; LOCF, Last observation carried forward*.

**Table 3:** Comparison of the VAS of gastrointestinal symptoms and stress of participants between the baseline and endpoint.

	**Baseline (*****n*** **=** **36)**		**8 weeks (*****n*** **=** **36; LOCF)**		**Mean differences (Endpoint-baseline)**		*p*-value^*^
	**Mean**	**SD**	**Mean**	**SD**	**Mean differences**	**SD**	
**VAS of gastrointestinal symptoms**							
Total	24.47	16.65	16.28	11.96	−8.19	12.91	0.001
Dry mouth	4.28	2.89	3.42	2.62	−0.86	2.71	0.064
Difficulty swallowing	1.17	1.77	1.08	2.09	−0.08	1.61	0.758
Decreased appetite	2.50	2.58	1.33	1.79	−1.17	2.85	0.019
Nausea or vomiting	1.08	1.65	0.75	1.34	−0.33	1.37	0.154
Flatulence	3.69	2.85	2.56	2.71	−1.14	2.77	0.019
Gastralgia	2.78	3.21	1.94	2.63	−0.83	2.29	0.036
Upper abdominal pain	1.61	2.36	0.81	1.51	−0.81	1.95	0.018
Lower abdominal pain	1.72	2.29	0.97	1.56	−0.75	2.03	0.034
Constipation	2.86	3.07	2.06	2.37	−0.86	1.79	0.010
Diarrhea	2.78	2.96	1.36	1.69	−1.42	2.57	0.002
VAS of stress	6.44	1.61	5.22	1.74	−1.22	1.84	<0.001

* *Changes between the baseline and endpoint were assessed by a paired t-test*.

*VAS, Visual Analog Scale*.

### Objective Outcomes

**Figure 2:**
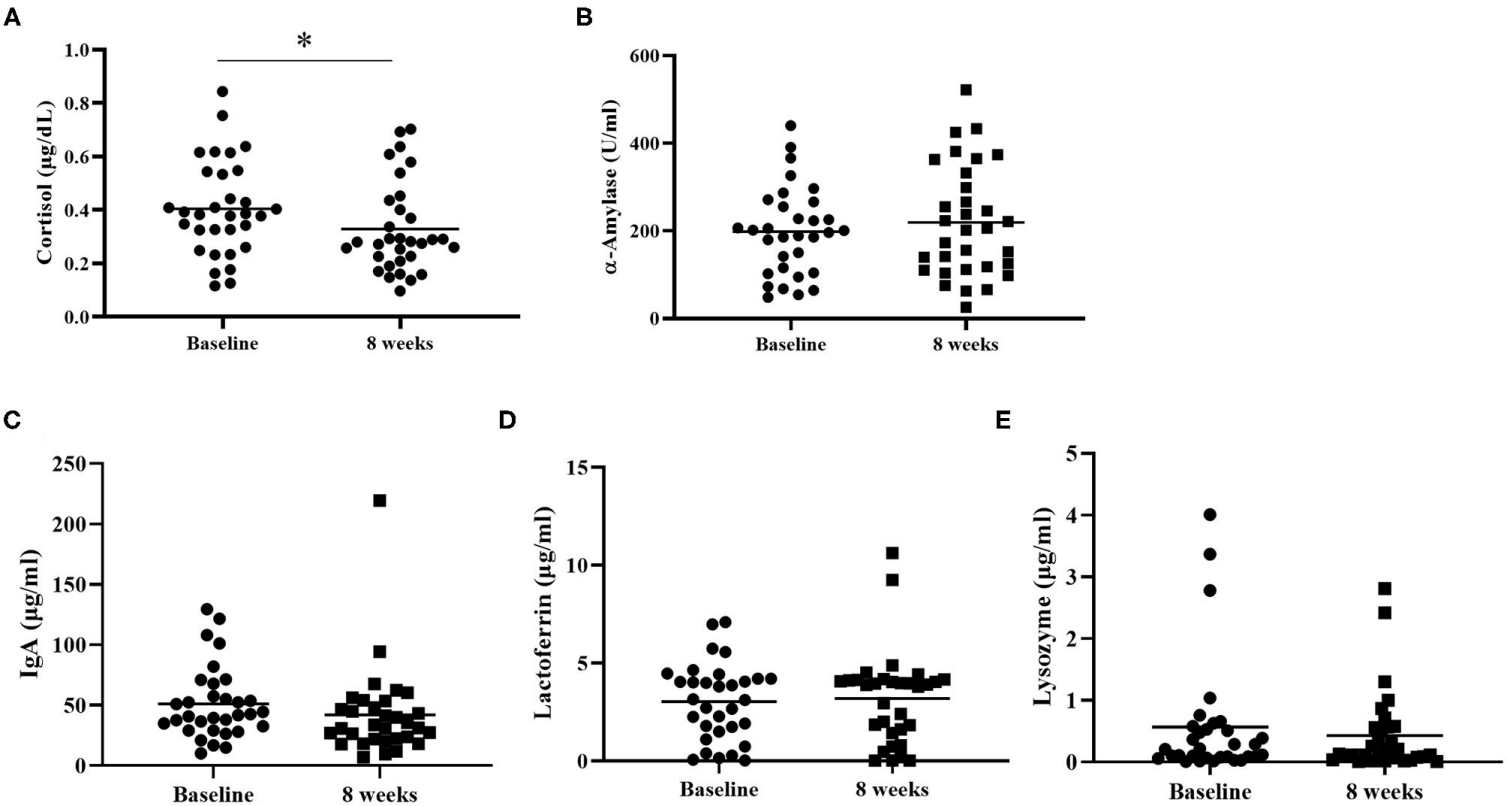
Salivary biomarkers. The levels of salivary cortisol **(A)**, α-amylase **(B)**, IgA **(C)**, lactoferrin **(D)**, and lysozyme **(E)** were determined before and after the 8-week intervention. *p < 0.05.

Table 4 shows the TAP results before and after the trial. No significant differences in flexibility, Go/No go, or working memory were found. Comparison of salivary stress biomarkers before and after the trial are shown in Figure 2. Cortisol levels were significantly decreased after the 8-week intervention period (*p* < 0.05). All other stress- and anxiety-related biomarkers were not significantly altered after the intervention.

**Table 4:** Comparison of the TAP results (seconds) of participants between the baseline and endpoint.

	**Baseline (*****n*** **=** **36)**		**8 weeks (*****n*** **=** **36; LOCF)**		**Mean differences (Endpoint-baseline)**		*p*-value^*^
	**Mean**	**SD**	**Mean**	**SD**	**Mean differences**	**SD**	
**TAP**							
Flexibility letter total	439.44	73.52	442.44	72.26	3.00	85.39	0.834
Flexibility number total	451.56	77.19	448.89	78.30	−2.67	85.49	0.853
Go/No go	406.64	66.25	409.06	60.84	2.42	48.96	0.769
Working memory	686.31	122.12	688.92	145.13	2.61	111.41	0.889

* *Changes between the baseline and endpoint were assessed by a paired t-test*.

*TAP, Tests of Attentional Performance*.

## Discussion

This is the first study to evaluate the effectiveness of the psychobiotic, PS128^TM^, on stress and the psychological well-being of highly stressed IT specialists. Our findings revealed that PS128^TM^ administration improved self-perceived stress, overall job-related stress, job burden, cortisol levels, general or physical health, anxiety, depression, sleep disturbances, quality of life, and both positive and negative emotions after the 8-week PS128^TM^ trial period. These outcomes suggest that PS128^TM^ has the distinct advantage of providing stress relief and improving mental health in employees under high work-related stress.

The decrease in PSS scores by more than 20% compared to baseline indicated that PS128^TM^ may play a role in sensitivity and perception of pressure in highly stressed workers. This result is in line with previous research describing a significant decrease in stress symptoms after probiotic intake (54, 55), but in contrast with another finding that there was no change in stress over time among healthy volunteers (20). This disparity may be due to the heterogeneity of the study population or differences in the probiotic strains administered. Few studies have focused on the possible effects of stress regulation by probiotics in the subclinical population of highly stressed workers, and the biological mechanisms remain to be established. It is probable that probiotics affect the central nervous system (CNS) while maintaining neuronal development through the neuroendocrine system, immune system, vagus nerves, neurotransmitters, and their receptors (13). From the findings of rodent models, we may speculate that changes in emotions and sensations in response to stress were associated with alterations in vagal afferent signaling, or by systemic metabolic changes associated with amino acids and polysaccharides (56, 57). Elucidating the underlying mechanism still requires further investigation by assessing larger controlled samples.

The significant decrease in depressive symptoms and negative emotions observed in our study were in agreement with previous studies reporting small, but significant reductions in depression (17). Furthermore, the significant decreases in both state and trait anxiety after probiotic administration were similar to those of a previous study that revealed significant reductions in anxiety scores among pregnant and postpartum women (18), but in contrast with other reports of non-significant differences after probiotic use in healthy volunteers with anxiety symptoms (17). This inconsistency may be because this study assessed highly stressed IT specialists who may have already had some subclinical psychological burden, whereas most previous research excluded participants with psychiatric conditions that required clinical attention, and included community samples with no or only low levels of anxiety that did not reach clinical diagnosis (17). From our results, it remains to be elucidated whether the alleviation of perceived stress was secondary to positive improvements in depression, anxiety, insomnia, and positive or negative emotions, or vice versa. Of note, improvements in perceived quality of life and positive emotions of “happiness” and “hopefulness” may suggest explanations other than the aspect of resilience, which differs from the traditional disease treatment model. In particular, we found a significant correlation between the decrease in cortisol level and increase in positive emotions. It has been suggested that possible mechanisms for the positive effects of probiotics on the CNS may include their ability to regulate mood or emotions by influencing the HPA axis, altering neural signaling pathways, or CNS neurotransmitters levels of serotonin and gamma-aminobutyric acid (GABA) (58, 59), or regulating inflammatory or immune responses by the gut microbiota (60). However, we did not find significant correlations between changes in salivary cortisol levels and changes in depression, anxiety, or perceived stress. Although this might indicate that the main mechanisms by which PS128^TM^ alleviates depression or anxiety may not be strongly associated with the HPA axis, other possibilities of decreased intestinal permeability or anti-inflammatory effects by bacterial colonization still need to be taken into consideration (61). Further studies with larger sample sizes, placebo comparisons, and examination of inflammatory or neuroendocrinal biomarkers are needed to clarify whether the improvements may be due to adaptive immune, metabolic, or neural pathways.

Our finding that the reduction in insomnia severity was complemented by the decrease in salivary cortisol levels, which might indicate possible correlations between probiotic use and sleep improvements through the HPA axis. This finding was in concordance with studies that have shown that some psychobiotics have sleep-improving effects in mice (62) or humans (63, 64), but in contrast with another study that found that the psychobiotic strain JB-1 did not improve sleep compared to the placebo in healthy male volunteers (19). Previous studies have shown that cortisol may inhibit the syntheses of interleukin-1beta (IL-1β) or tumor necrosis factor alpha (TNF-α) (65). Presence of these cytokines in the human blood were found to influence the circadian rhythm and nonrapid eye movements (nREM) sleep. A weak immune response against bacterial cell wall components of PS128^TM^ may help decrease cortisol and increase cytokine secretion (21, 22, 66). Such changes may contribute to more nREM sleep and better sleep quality. However, excessive cytokines might still disturb the sleep structure (61, 67). Other proposed explanations for the sleep-improving effects of PS128^TM^ on the regulation of wakefulness or sleep might be related to several neurotransmitters, such as the increase in GABA (68), decrease in orexin levels or increase in inhibitory adenosine levels in the ventrolateral preoptic nucleus, striatum, hypothalamus, hippocampus, and prefrontal cortex (62, 69). Other non-invasive methods for monitoring changes in sleep stages may be required to clarify how PS128^TM^ affects the sleep structure among highly stressed individuals.

We found that salivary cortisol levels decreased after the 8-week probiotics trial, but other biomarkers, such as α-amylase, IgA, lactoferrin, or lysozyme did not significantly deviate from baseline levels. Similarly, no significant differences were noted when comparing the cognitive performance such as attention and working memory between the baseline and endpoint. α-Amylase was found to be a surrogate marker for sympathetic activities (70). Our findings may indicate that improvements in perceived stress or mood symptoms were not specifically associated with acute adrenergic responses. The lack of a significant change in objective measures of stress, including IgA, lactoferrin, lysozyme, or attentional performance after probiotic treatment might indicate other underlying mechanisms that we were unable to determine in the current study. Explanations for our findings may be restricted by the small sample size and the open-label design, and future investigations that assess more objective biological plasma biomarkers or brain imaging studies are warranted.

The major strength of this study is the use of well-validated stress, psychological, or salivary measures to extensively assess the relevant aspects. Key limitations include the small sample size and open-label design. Further larger-scale randomized, placebo-controlled studies may be needed to demonstrate the effectiveness of PS128^TM^ against stress. Second, although we specifically recruited participants with high perceived stress, our study sample may only represent highly stressed workers in the IT industry. Extension of the generalizability to other occupations may be restricted. Despite that our results from this single arm pilot study provided further justification on the safety and tolerability profiles of PS128^TM^, owing to the above limitations, sub-project 1, the four-arm randomized controlled trial among high-stress registered nurses is currently undergoing. We aimed that results from this double blind, placebo-controlled trial with larger sample size of at least 60 people in each arm may be powered to overcome these shortcomings. Third, although we assessed many self-reported psychological conditions, objective evaluations of mental health state or direct examinations of physiological or psychological responses under acute stressors may further extend our understanding of probiotics and stress-induced reactions.

Our study demonstrated that oral supplementation with PS128^TM^ may improve perceived levels of overall or job-related stress, as well as mental health states among highly stressed IT specialists. Good tolerability and safety profiles have also been revealed. As for the direction of future research, in addition to conducting randomized controlled trials with larger sample sizes, investigations on gut permeability, types of gastrointestinal microbiota, production rates of short chain fatty acids, brain images for functional or structural changes, and other biological markers associated with the HPA axis or inflammation such as cytokines may also help elucidate possible mechanism of action of probiotics in highly stressed specialists.

## Data Availability Statement

The original contributions presented in the study are included in the article/supplementary material, further inquiries can be directed to the corresponding author/s.

## Ethics Statement

The studies involving human participants were reviewed and approved by Institutional Review Board of Mackay Memorial Hospital (IRB no: 19CT013be). The patients/participants provided their written informed consent to participate in this study.

## Author Contributions

S-IW, C-CW, and P-JT conceived and designed study. C-CH and I-KS assisted with the methodology. S-IW and W-LC performed the investigation. S-IW, P-YC, L-HC, T-WL, C-JK, and W-LC curated and analyzed the data. S-IW interpreted results and drafted the manuscript. S-IW, C-CW, P-YC, and C-JK reviewed and edited the manuscript. Y-CT supervised the study. All authors have read and agreed to the published version of the manuscript.

## Conflict of Interest

C-CW, P-JT, L-HC, and C-CH are employees of Bened Biomedical Co., Ltd. Y-CT owns stock in Bened Biomedical Co., Ltd. The views of this article reflect those of the authors and not necessarily those of the funder. The remaining authors declare that the research was conducted in the absence of any commercial or financial relationships that could be construed as a potential conflict of interest.

## Abbreviations

IT: Information technology
PSS: Perceived Stress Scale
STAI: State Trait Anxiety Index
JSS: Job Stress Scale
ISI: Insomnia Severity Index
PHQ: Patient Health Questionnaire
QLESQ: Quality of Life Enjoyment and Satisfaction Questionnaire
LOCF: Last observation carried forward
VAS: Visual Analog Scale
TAP: Tests of Attentional Performance
nREM: nonrapid eye movements.

## References

[1] Leavitt HJ Whisler T.L. Management in the 1980s. Harvard Bus Rev. (1958) 11. Available online at: https://hbr.org/1958/11/management-in-the-1980s (accessed March 17, 2021).

[2] Csorny L. Careers in the Growing Field of Information Technology Services. Beyond the Numbers, Employment and Unemployment, U.S. Bureau of Labor Statistics (2013) (cited 2020).

[3] Optronics A. The History of Taiwan Information Technology Business (2013).

[4] National Statistics ROCT. Employments by Industry, 10th Revision, 2017~2020. (2020) (cited 2020).

[5] Wallgren LG Hanse JJ. Job characteristics, motivators and stress among information technology consultants: a structural equation modeling approach. Int J Ind Ergon. (2007) 37:51–9. 10.1016/j.ergon.2006.10.005

[6] Padma V Anand NN Gurukul SMGS Javid SMASM Prasad A Arun S. Health problems and stress in Information Technology and Business Process Outsourcing employees. J Pharm Bioallied Sci. (2015) 7(Suppl 1):S9–S13. 10.4103/0975-7406.155764 26015763 PMC4439723

[7] Kalmbach DA Anderson JR Drake CL. The impact of stress on sleep: pathogenic sleep reactivity as a vulnerability to insomnia and circadian disorders. J Sleep Res. (2018) 27:e12710. 10.1111/jsr.12710 29797753 PMC7045300

[8] Lin SH Liao WC Chen MY Fan JY. The impact of shift work on nurses' job stress, sleep quality and self-perceived health status. J Nurs Manag. (2014) 22:604–12. 10.1111/jonm.12020 25041800

[9] Chin W Guo YL Hung YJ Yang CY Shiao JS. Short sleep duration is dose-dependently related to job strain and burnout in nurses: a cross sectional survey. Int J Nurs Stud. (2015) 52:297–306. 10.1016/j.ijnurstu.2014.09.003 25311378

[10] Sang E Liao YM Miao NF Chou KR Chung MH. Patterns and correlates of benzodiazepine use in nurses: a nationwide, population-based study. Int J Mental Health Nurs. (2018) 27:400–7. 10.1111/inm.12334 28374978

[11] Reynolds AC Paterson JL Ferguson SA Stanley D Wright KP Jr Dawson D. The shift work and health research agenda: considering changes in gut microbiota as a pathway linking shift work, sleep loss and circadian misalignment, and metabolic disease. Sleep Med Rev. (2017) 34:3–9. 10.1016/j.smrv.2016.06.009 27568341

[12] Clemente JC Ursell LK Parfrey LW Knight R. The impact of the gut microbiota on human health: an integrative view. Cell. (2012) 148:1258–70. 10.1016/j.cell.2012.01.035 22424233 PMC5050011

[13] Collins SM Surette M Bercik P. The interplay between the intestinal microbiota and the brain. Nat Rev Microbiol. (2012) 10:735–42. 10.1038/nrmicro2876 23000955

[14] Desbonnet L Garrett L Clarke G Kiely B Cryan JF Dinan TG. Effects of the probiotic Bifidobacterium infantis in the maternal separation model of depression. Neuroscience. (2010) 170:1179–88. 10.1016/j.neuroscience.2010.08.005 20696216

[15] De Palma G Blennerhassett P Lu J Deng Y Park A Green W. Microbiota and host determinants of behavioural phenotype in maternally separated mice. Nat Commun. (2015) 6:7735. 10.1038/ncomms8735 26218677

[16] Dinan TG Stanton C Cryan JF. Psychobiotics: a novel class of psychotropic. Biol Psychiatry. (2013) 74:720–6. 10.1016/j.biopsych.2013.05.001 23759244

[17] Liu RT Walsh RFL Sheehan AE. Prebiotics and probiotics for depression and anxiety: a systematic review and meta-analysis of controlled clinical trials. Neurosci Biobehav Rev. (2019) 102:13–23. 10.1016/j.neubiorev.2019.03.023 31004628 PMC6584030

[18] Slykerman RF Hood F Wickens K Thompson JMD Barthow C Murphy R. Effect of *Lactobacillus rhamnosus* HN001 in pregnancy on postpartum symptoms of depression and anxiety: a randomised double-blind placebo-controlled trial. EBioMedicine. (2017) 24:159–65. 10.1016/j.ebiom.2017.09.013 28943228 PMC5652021

[19] Kelly JR Allen AP Temko A Hutch W Kennedy PJ Farid N. Lost in translation? The potential psychobiotic Lactobacillus rhamnosus (JB-1) fails to modulate stress or cognitive performance in healthy male subjects. Brain Behav Immun. (2017) 61:50–9. 10.1016/j.bbi.2016.11.018 27865949

[20] Messaoudi M Lalonde R Violle N Javelot H Desor D Nejdi A. Assessment of psychotropic-like properties of a probiotic formulation (*Lactobacillus helveticus* R0052 and *Bifidobacterium longum* R0175) in rats and human subjects. Br J Nutr. (2011) 105:755–64. 10.1017/S0007114510004319 20974015

[21] Liu WH Yang CH Lin CT Li SW Cheng WS Jiang YP. Genome architecture of *Lactobacillus plantarum* PS128, a probiotic strain with potential immunomodulatory activity. Gut Pathogens. (2015) 7:22. 10.1186/s13099-015-0068-y 26279684 PMC4536865

[22] Liu YW Liu WH Wu CC Juan YC Wu YC Tsai HP. Psychotropic effects of *Lactobacillus plantarum* PS128 in early life-stressed and naive adult mice. Brain Res. (2016) 1631:1–12. 10.1016/j.brainres.2015.11.018 26620542

[23] Liu WH Chuang HL Huang YT Wu CC Chou GT Wang S. Alteration of behavior and monoamine levels attributable to Lactobacillus plantarum PS128 in germ-free mice. Behav Brain Res. (2016) 298(Pt B):202–9. 10.1016/j.bbr.2015.10.046 26522841

[24] Liao JF Cheng YF Li SW Lee WT Hsu CC Wu CC. *Lactobacillus plantarum* PS128 ameliorates 2,5-Dimethoxy-4-iodoamphetamine-induced tic-like behaviors via its influences on the microbiota-gut-brain-axis. Brain Res Bull. (2019) 153:59–73. 10.1016/j.brainresbull.2019.07.027 31351942

[25] Liu YW Wang YP Yen HF Liu PY Tzeng WJ Tsai CF. *Lactobacillus plantarum*. PS128 ameliorated visceral hypersensitivity in rats through the gut-brain axis. Probiotics Antimicrob Proteins. (2019) 12:980–93. 10.1007/s12602-019-09595-w 31691208

[26] Liu YW Liong MT Chung YE Huang HY Peng WS Cheng YF. Effects of *Lactobacillus plantarum* PS128 on children with autism spectrum disorder in Taiwan: a randomized, double-blind, placebo-controlled trial. Nutrients. (2019) 11:820. 10.3390/nu11040820 30979038 PMC6521002

[27] Huang WC Wei CC Huang CC Chen WL Huang HY. The Beneficial effects of *Lactobacillus plantarum* PS128 on high-intensity, exercise-induced oxidative stress, inflammation, and performance in triathletes. Nutrients. (2019) 11:353. 10.3390/nu11020353 30736479 PMC6412901

[28] Chang JS Chiu YH Pan CC Chen CH. Probiotics *Lactobacillus plantarum* PS128 intervention in two patients with major depressive disorder. Taiwan J Psychiatry. (2019) 33:116–7. 10.4103/TPSY.TPSY_22_19

[29] Liao PL Wu CC Chen TY Tsai YC Peng WS Yang DJ. Toxicity studies of *Lactobacillus plantarum* PS128TM isolated from spontaneously fermented mustard greens. Foods. (2019) 8:668. 10.3390/foods8120668 PMC6963738 31835837

[30] Cohen S Kamarck T Mermelstein R. A global measure of perceived stress. J Health Soc Behav. (1983) 24:385–96. 10.2307/2136404 6668417

[31] Robles Z Garey L Hogan J Bakhshaie J Schmidt NB Zvolensky MJ. Examining an underlying mechanism between perceived stress and smoking cessation-related outcomes. Addict Behav. (2016) 58:149–54. 10.1016/j.addbeh.2016.02.022 26946445 PMC5531612

[32] Bhandary B Rao S T SS. The effect of perceived stress and family functioning on people with type 2 diabetes mellitus. J Clin Diagn Res. (2013) 7:2929–31. 10.7860/JCDR/2013/7414.3689 24551677 PMC3919319

[33] Ezzati A Jiang J Katz MJ Sliwinski MJ Zimmerman ME Lipton RB. Validation of the Perceived Stress Scale in a community sample of older adults. Int J Geriatr Psychiatry. (2014) 29:645–52. 10.1002/gps.4049 24302253 PMC4013212

[34] Baik SH Fox RS Mills SD Roesch SC Sadler GR Klonoff EA. Reliability and validity of the Perceived Stress Scale-10 in Hispanic Americans with English or Spanish language preference. J Health Psychol. (2019) 24:628–39. 10.1177/1359105316684938 28810432 PMC6261792

[35] Chu LC Kao HSR. The moderation of meditation experience and emotional intelligence on the relationship between perceived stress and negative mental health. Chin J Psychol. (2005) 47:157–79. 10.6129/CJP.2005.4702.05

[36] Spielberger CD Gorsuch RL Lushene R Vagg PR Jacobs GA. Manual for the State-Trait Anxiety Inventory. Palo Alto, CA: Consulting Psychologists Press (1983).

[37] Shek DT. The Chinese version of the State-Trait Anxiety Inventory: its relationship to different measures of psychological well-being. J Clin Psychol. (1993) 49:349–58. 10.1002/1097-4679(199305)49:3<349::AID-JCLP2270490308>3.0.CO;2-J 8315037

[38] Chang PJ Yeh YC. The Relationships between Gender, Birth Order, Family Structure, Emotion, Creative Personalities and Technological Creativity of Fifth Graders. National Chung-Shan University (2003).

[39] Liu SI Yeh ZT Huang HC Sun FJ Tjung JJ Hwang LC. Validation of patient health questionnaire for depression screening among primary care patients in Taiwan. Compr Psychiatry. (2011) 52:96–101. 10.1016/j.comppsych.2010.04.013 21111406

[40] Gagnon C Bélanger L Ivers H Morin CM. Validation of the insomnia severity index in primary care. J Am Board Fam Med. (2013) 26:701–10. 10.3122/jabfm.2013.06.130064 24204066

[41] Endicott J Nee J Harrison W Blumenthal R. Quality of Life Enjoyment and Satisfaction Questionnaire: a new measure. Psychopharmacol Bull. (1993) 29:321–6. 10.1037/t49981-000 8290681

[42] Lee YT Liu SI Huang HC Sun FJ Huang CR Yeung A. Validity and reliability of the Chinese version of the Short Form of Quality of Life Enjoyment and Satisfaction Questionnaire (Q-LES-Q-SF). Qual Life Res. (2014) 23:907–16. 10.1007/s11136-013-0528-0 24062242

[43] Cooper CL. Identifying stressors at work: recent research developments. J Psychosom Res. (1983) 27:369–76. 10.1016/0022-3999(83)90068-5 6668563

[44] The Ministry of Labor: The Job Stress Scale (cited 2018).

[45] Schneider CK Melmed RD Barstow LE Enriquez FJ Ranger-Moore J Ostrem JA. Oral human immunoglobulin for children with autism and gastrointestinal dysfunction: a prospective, open-label study. J Autism Dev Disord. (2006) 36:1053–64. 10.1007/s10803-006-0141-y 16845577

[46] Diop L Guillou S Durand H. Probiotic food supplement reduces stress-induced gastrointestinal symptoms in volunteers: a double-blind, placebo-controlled, randomized trial. Nutr Res. (2008) 28:1–5. 10.1016/j.nutres.2007.10.001 19083380

[47] Lesage FX Berjot S. Validity of occupational stress assessment using a visual analogue scale. Occup Med. (2011) 61:434–6. 10.1093/occmed/kqr037 21505089

[48] Psytest-TAP 2.3.1 (cited 2018).

[49] Fortier-Brochu E Beaulieu-Bonneau S Ivers H Morin CM. Insomnia and daytime cognitive performance: a meta-analysis. Sleep Med Rev. (2012) 16:83–94. 10.1016/j.smrv.2011.03.008 21636297

[50] Iizuka N Awano S Ansai T. Salivary alpha-amylase activity and stress in Japan air self-defense force cargo pilots involved in Iraq reconstruction. Am J Hum Biol. (2012) 24:468–72. 10.1002/ajhb.22247 22344628

[51] Yang Y Koh D Ng V Lee CY Chan G Dong F. Self perceived work related stress and the relation with salivary IgA and lysozyme among emergency department nurses. Occup Environ Med. (2002) 59:836–41. 10.1136/oem.59.12.836 12468751 PMC1763606

[52] Shinjo T Sakuraba K Nakaniida A Ishibashi T Kobayashi M Aono Y. Oral lactoferrin influences psychological stress in humans: a single-dose administration crossover study. Biomed Rep. (2018) 8:426–32. 10.3892/br.2018.1076 29850019 PMC5962838

[53] Lalitsuradej E Sivamaruthi B Sirilun S Sittiprapaporn P Peerajan S Chaiyasut C. The effect of supplementation of *Lactobacillus paracasei* HII01 on salivary cortisol, and dehydroepiandrosterone sulfate (DHEA-S) levels. Asian J Med Sci. (2020) 11:12–5. 10.3126/ajms.v11i1.26500

[54] Mohammadi AA Jazayeri S Khosravi-Darani K Solati Z Mohammadpour N Asemi Z. The effects of probiotics on mental health and hypothalamic-pituitary-adrenal axis: a randomized, double-blind, placebo-controlled trial in petrochemical workers. Nutr Neurosci. (2016) 19:387–95. 10.1179/1476830515Y.0000000023 25879690

[55] Allen AP Hutch W Borre YE Kennedy PJ Temko A Boylan G. *Bifidobacterium longum* 1714 as a translational psychobiotic: modulation of stress, electrophysiology and neurocognition in healthy volunteers. Transl Psychiatry. (2016) 6:e939. 10.1038/tp.2016.191 27801892 PMC5314114

[56] Tillisch K Labus J Kilpatrick L Jiang Z Stains J Ebrat B. Consumption of fermented milk product with probiotic modulates brain activity. Gastroenterology. (2013) 144:1394–401.e1–4. 10.1053/j.gastro.2013.02.043 23474283 PMC3839572

[57] Nicholson JK Holmes E Kinross J Burcelin R Gibson G Jia W. Host-gut microbiota metabolic interactions. Science. (2012) 336:1262–7. 10.1126/science.1223813 22674330

[58] Carding S Verbeke K Vipond DT Corfe BM Owen LJ. Dysbiosis of the gut microbiota in disease. Microb Ecol Health Dis. (2015) 26:26191. 10.3402/mehd.v26.26191 25651997 PMC4315779

[59] Foster JA McVey Neufeld KA. Gut-brain axis: how the microbiome influences anxiety and depression. Trends Neurosci. (2013) 36:305–12. 10.1016/j.tins.2013.01.005 23384445

[60] Josipa Vlainić V Jelena Š Toni V Antonella Letizia V. Probiotics as an adjuvant therapy in major depressive disorder. Curr Neuropharmacol. (2016) 14:952–8. 10.2174/1570159X14666160526120928 27226112 PMC5333591

[61] Galland L. The gut microbiome and the brain. J Med Food. (2014) 17:1261–72. 10.1089/jmf.2014.7000 25402818 PMC4259177

[62] Lin A Shih CT Huang CL Wu CC Lin CT Tsai YC. Hypnotic effects of *Lactobacillus fermentum* PS150(TM) on pentobarbital-induced sleep in mice. Nutrients. (2019) 11:2409. 10.3390/nu11102409 31600934 PMC6836230

[63] Nishida K Sawada D Kawai T Kuwano Y Fujiwara S Rokutan K. Para-psychobiotic Lactobacillus gasseri CP2305 ameliorates stress-related symptoms and sleep quality. J Appl Microbiol. (2017) 123:1561–70. 10.1111/jam.13594 28948675

[64] Takada M Nishida K Kataoka-Kato A Gondo Y Ishikawa H Suda K. Probiotic *Lactobacillus casei* strain Shirota relieves stress-associated symptoms by modulating the gut-brain interaction in human and animal models. Neurogastroenterol Motil. (2016) 28:1027–36. 10.1111/nmo.12804 26896291

[65] Schuld A Haack M Hinze-Selch D Mullington J Pollmacher T. [Experimental studies on the interaction between sleep and the immune system in humans]. Psychother Psychosom Med Psychol. (2005) 55:29–35. 10.1055/s-2004-834561 15647993

[66] Li Y Hao Y Fan F Zhang B. The role of microbiome in insomnia, circadian disturbance and depression. Front Psychiatry. (2018) 9:669. 10.3389/fpsyt.2018.00669 30568608 PMC6290721

[67] Cermakian N Lange T Golombek D Sarkar D Nakao A Shibata S. Crosstalk between the circadian clock circuitry and the immune system. Chronobiol Int. (2013) 30:870–88. 10.3109/07420528.2013.782315 23697902 PMC7195843

[68] Higo-Yamamoto S Yamamoto S Miyazaki K Nakakita Y Kaneda H Takata Y. Dietary heat-killed *Lactobacillus brevis* SBC8803 attenuates chronic sleep disorders induced by psychophysiological stress in mice. J Nutr Sci Vitaminol (Tokyo). (2019) 65:164–70. 10.3177/jnsv.65.164 31061285

[69] Krystal AD Benca RM Kilduff TS. Understanding the sleep-wake cycle: sleep, insomnia, and the orexin system. J Clin Psychiatry. (2013) 74(Suppl 1):3–20. 10.4088/JCP.13011su1c 24107804

[70] Petrakova L Doering BK Vits S Engler H Rief W Schedlowski M. Psychosocial stress increases salivary alpha-amylase activity independently from plasma noradrenaline levels. PLoS One. (2015) 10:e0134561. 10.1371/journal.pone.0134561 26247781 PMC4527714

